# IMMUNE RESPONSES TO MODERNA COVID-19 MRNA VACCINE IN FRAIL NURSING HOME PATIENTS

**DOI:** 10.1093/geroni/igac059.1794

**Published:** 2022-12-20

**Authors:** Charles Semelka, John Sanders, Martha Alexander-Miller

**Affiliations:** Wake Forest School of Medicine, Winston Salem, North Carolina, United States; Wake Forest School of Medicine, Winston Salem, North Carolina, United States; Wake Forest School of Medicine, Winston-Salem, North Carolina, United States

## Abstract

Prior COVID-19 mRNA vaccine trials included healthy older adults, but mRNA vaccine responses were not studied in frail older adults. We postulated that frailty was associated with immune responses of reduced quality and quantity following mRNA vaccination. A cohort of 15 older adults in a retirement facility were followed from the first Moderna mRNA-1273 vaccine dose in February 2021 with blood collections at baseline and weeks 4 (boost), 6, 18 and 28. Outcomes were IgG titers to SARS-CoV-2 Spike protein with secondary outcomes of T cell responses. Statistical analysis used log transformed geometric mean antibody titers in multivariable regression models with clinical predictors including, age, sex, prior infection status, and clinical frailty scale (CFS) score. Cellular immune response analysis used multivariable regression for function and phenotyping of T cell subsets. All participants with median (IQR) age: 90 years (84, 96) and CFS score: moderately frail 6 (5, 7) generated robust antibody responses with mean peak titer levels 10-fold higher than baseline. In the adjusted model, individuals with severely frail scores CFS=7 had lower antibody levels compared to mildly frail CFS=5, OR: 0.55 (0.35, 0.87) p=0.017. Both chronological age and sex had non-significant relationships with antibody titers. Spike peptide specific CD4 cells and T follicular helper cells were significantly decreased in more frail individuals (p=0.011 and p=0.008 respectively), though the relationship with antibody titers was non-significant. Frailty scores were a better predictor than age for serologic and cellular immune responses to COVID-19 mRNA vaccination in very old adults.

